# Retraction: Ppm1b Negatively Regulates 3-Bromopyruvic Acid Induced Necroptosis in Breast Cancer Cells

**DOI:** 10.3389/fonc.2021.757083

**Published:** 2021-08-26

**Authors:** Frontiers Editorial Office

**Affiliations:** Frontiers Media SA, Lausanne, Switzerland

The Journal and Authors retract the 14 January 2021 article cited above for the following reasons provided by the Authors:

Following publication, concerns were raised regarding the integrity of the images in the published figures. The authors failed to provide a satisfactory explanation during the investigation, which was conducted in accordance with Frontiers’ policies.

This retraction was approved by the Chief Editors of Frontiers in Oncology and the Chief Executive Editor of Frontiers. The authors agree to this retraction.

